# The Hunt Is On! In Pursuit of the Ideal Stem Cell Population for Cartilage Regeneration

**DOI:** 10.3389/fbioe.2022.866148

**Published:** 2022-05-27

**Authors:** T. Mark Campbell, F. Jeffrey Dilworth, David S. Allan, Guy Trudel

**Affiliations:** ^1^ Elisabeth Bruyère Hospital, Ottawa, ON, Canada; ^2^ Bone and Joint Research Laboratory, Ottawa Hospital Research Institute, Ottawa, ON, Canada; ^3^ Regenerative Medicine, Ottawa Hospital Research Institute, Ottawa, ON, Canada; ^4^ Department of Medicine, The Ottawa Hospital, Ottawa, ON, Canada; ^5^ Department of Cellular and Molecular Medicine, Faculty of Medicine, University of Ottawa, Ottawa, ON, Canada; ^6^ Department of Biochemistry, Immunology and Microbiology, Faculty of Medicine, University of Ottawa, Ottawa, ON, Canada

**Keywords:** cartilage, regenerative medicine, growth plate, osteoarthritis, musculoskeletal health, stem cells

## Abstract

Cartilage injury and degeneration are hallmarks of osteoarthritis (OA), the most common joint disease. OA is a major contributor to pain, loss of function, and reduced quality of life. Over the last decade, considerable research efforts have focused on cell-based therapies, including several stem cell-derived approaches to reverse the cartilage alterations associated with OA. Although several tissue sources for deriving cell-based therapies have been identified, none of the resident stem cell populations have adequately fulfilled the promise of curing OA. Indeed, many cell products do not contain true stem cells. As well, issues with aggressive marketing efforts, combined with a lack of evidence regarding efficacy, lead the several national regulatory bodies to discontinue the use of stem cell therapy for OA until more robust evidence becomes available. A review of the evidence is timely to address the status of cell-based cartilage regeneration. The promise of stem cell therapy is not new and has been used successfully to treat non-arthritic diseases, such as hematopoietic and muscle disorders. These fields of regenerative therapy have the advantage of a considerable foundation of knowledge in the area of stem cell repair mechanisms, the role of the stem cell niche, and niche-supporting cells. This foundation is lacking in the field of cartilage repair. So, where should we look for the ideal stem cell to regenerate cartilage? It has recently been discovered that cartilage itself may contain a population of SC-like progenitors. Other potential tissues include stem cell-rich dental pulp and the adolescent growth plate, the latter of which contains chondrocyte progenitors essential for producing the cartilage scaffold needed for bone growth. In this article, we review the progress on stem cell therapies for arthritic disorders, focusing on the various stem cell populations previously used for cartilage regeneration, successful cases of stem cell therapies in muscle and hemopoietic disorders, some of the reasons why these other fields have been successful (i.e., “lessons learned” to be applied to OA stem cell therapy), and finally, novel potential sources of stem cells for regenerating damaged cartilage *in vivo*.

## Introduction

Osteoarthritis (OA) is a major health concern, affecting more than 50% of adults over the age of 65 (Loeser, 2010). OA contributes significantly to pain, disability, and rising healthcare costs (Cross et al., 2014). The average annual cost per person afflicted with OA is as high as €23,000, a massive sum considering the millions of individuals affected with OA worldwide. In the United States, the annual cost of OA is >$16.5 billion, accounting for >4% of the combined costs for all hospitalizations (CDC Prevention, 2021). OA of the hip and knee contribute the most to OA burden, often resulting in joint replacement surgery, including >1 million annual joint replacements in the United States and roughly 1,60,000 in the United Kingdom (Centers for Disease Control and Prevention, 2010; Cross et al., 2014; Registry, 2020). It is predicted that OA will soon be the fourth most disabling chronic disease in the world, and OA is the fastest growing major health condition (Silverwood et al., 2015). The burden of OA has, therefore, become an urgent international healthcare issue.

OA is a total joint disease, but the end result is the complete loss of articular cartilage. The presence of early cartilage defects is a strong risk factor for OA progression (Guermazi et al., 2017; Everhart et al., 2019). In the OA-affected joint, the products of cartilage breakdown that are released into the synovial fluid are phagocytosed by synovial cells, amplifying synovial inflammation (Sellam and Berenbaum, 2010). In turn, activated synovial cells in the inflamed synovium produce catabolic and pro-inflammatory mediators, such as interleukins 1 and 6 and tumor necrosis factor-α (Sellam and Berenbaum, 2010). This inflammatory response is amplified by activated synovial T cells, B cells, and infiltrating macrophages (Sellam and Berenbaum, 2010). The resulting inflammatory milieu leads to the secretion of matrix-degrading enzymes from chondrocytes, further propagating tissue breakdown and creating a positive feedback loop as joint degeneration continues and progresses (Sellam and Berenbaum, 2010; Mata et al., 2017). To date, there are no accepted disease-modifying OA drugs (DMOADs) to slow OA progression; therefore, treatment has been aimed at reducing symptoms (McAlindon et al., 2014). Analgesic medications such as acetaminophen and nonsteroidal anti-inflammatory drugs do not alter OA-related degeneration. Though some studies evaluating cartilage-based treatment with nutritional supplements such as glucosamine and chondroitin sulfate have suggested these treatments reduce pain and delay in structural progression, other studies have shown equivocal results (Towheed et al., 2009; Bruyère et al., 2016), generating equipoise as to whether these nutritional supplements should be recommended (Hochberg et al., 2012; McAlindon et al., 2014). Antibodies targeting pain pathways, such as tanezumab, have shown some benefits in clinical trials but are believed to contribute to rapidly-progressive OA in a notable proportion of treated individuals, thus they are presently excluded from OA treatment guidelines (Schnitzer et al., 2019; Berenbaum et al., 2020). In addition to changes in cartilage, other articular tissues are affected by OA, including the synovium, ligament, and bone (Barr et al., 2015). Treatments, such as strontium ranelate, directed at preventing pathologic bone alterations have shown some positive effects on clinical outcomes such as pain and disability (Reginster et al., 2013; Bruyère et al., 2016), and possibly structural progression (Roubille et al., 2015); however, these results have not been widely accepted, nor has strontium ranelate been approved for OA treatment (Hochberg et al., 2012; McAlindon et al., 2014).

Surgical strategies to halt the progression from cartilage defect to the development of OA have been limited and prone to failure (Palmer et al., 2019; Zamborsky and Danisovic, 2020). Marrow-stimulation procedures, such as microfracture, rely on the development of a primitive mesenchymal blood clot that often forms fibrous tissue with variable patient outcomes (Haleem et al., 2010). Osteochondral grafting limitations include donor site availability, morbidity, and fibrocartilage formation between osteochondral plugs (Redman et al., 2005; Haleem et al., 2010). Once OA is established, treatment is essentially palliative (McAlindon et al., 2014). As such, research has turned to stem cell-based therapies to slow and/or reverse OA, an area of research that has expanded dramatically over the last decade (Koelling and Miosge, 2009; Yubo et al., 2017; Whittle et al., 2019; Zhang et al., 2019).

## Recruiting and Stimulating Endogenous Stem Cells to Treat Osteoarthritis

The development of OA in the joint represents a failure of the endogenous articular repair system to maintain healthy osteochondral units (Goldring and Goldring, 2016). Specific to articular cartilage, native chondrocytes are unable to maintain the extracellular matrix, resulting in cartilage fibrillation, fissuring, and thinning (Pritzker et al., 2006; Sulzbacher, 2013). Over time, cartilage may erode completely, exposing the subchondral bone (Moisio et al., 2009). A complete understanding of the mechanism(s) by which cartilage regeneration fails is lacking; however, the reparative function of the chondrocyte may be disrupted by pathologic changes in the OA joint microenvironment (Sellam and Berenbaum, 2010; Jayasuriya et al., 2016). These changes include increased inflammation along with the production of reactive oxygen species and pro-degradation proteins, including matrix metalloproteinases and ADAMTS (a disintegrin and metalloproteinase with thrombospondin motifs) (Sellam and Berenbaum, 2010; Jayasuriya et al., 2016). In addition, the subchondral bone becomes more permeable, allowing bone morphogenetic proteins (BMPs) and transforming growth factor β (TGF-β) to diffuse into cartilage from the bone, favoring the terminal differentiation of chondrocytes and osteophyte formation (Zhen et al., 2013; Jayasuriya et al., 2016). As a result, the OA joint microenvironment becomes catabolic, with little support for endogenous cartilage repair. Biomechanical influences also play a role, with overloaded joint compartments experiencing accelerated OA-related structural changes such as osteophyte formation, bone attrition, and deformity, as well as microenvironmental OA alterations and greater overall susceptibility to cartilage injury and loss (Sharma et al., 2001; Guilak, 2011; Chen et al., 2013). Under such circumstances, additional aid must arrive at the site of damage to assist the failing chondrocytes. Mesenchymal stem cells (MSCs) have been proposed as strong candidates for enhancing the articular repair process (McGonagle et al., 2017). MSCs are clonogenic progenitor cells capable of differentiating into mesoderm-derived cells such as osteoblasts, chondrocytes, and adipocytes (Prockop, 1997). One attractive feature of MSCs is that they are found in many tissues of the synovial joints including the bone, synovium, and adipose, representing about 1% of the total cell population (Jones et al., 2010a). As well, endogenous MSCs play a supportive role in the immune system, providing immunomodulation that can either enhance or dampen the inflammatory cascade of the OA articular milieu by adapting their immunoregulatory properties to the local immunological environment (Hoogduijn, 2015; Song et al., 2020a). Through the excretion of cytokines, growth factors, chemokines, and cell–cell contact, MSCs exert their immunomodulatory effect on immune cells, such as T and B cells, natural killer (NK) cells, macrophages, monocytes, dendritic cells (DCs), and neutrophils, thus exerting a potentially potent effect on the local immune response (Song et al., 2020a). With this in mind, exogenous MSCs have been used to treat inflammatory conditions such as graft-versus-host disease, graft rejection, and autoimmune diseases (Müller et al., 2021). To our knowledge, however, capitalizing on the immunomodulatory capabilities of joint-resident MSCs has not yet been attempted for the treatment of OA.

MSCs were shown to accumulate in greater numbers in the regions of the damaged OA bone (Campbell et al., 2016). Directing such subchondral MSC populations to effectively restore the joint microenvironment and repair OA-associated cartilage damage would present a powerful therapeutic target to slow or halt OA progression, particularly if initiated early in the disease (McGonagle et al., 2017). McGonagle and Jones reviewed the potential origins of such reparative MSCs, noting that the MSC native environment of origin (niche) is critical to their function (McGonagle et al., 2017). As an example, synovium-derived MSCs showed superior chondrogenic potential as compared to those derived from bone or subcutaneous fat (Sakaguchi et al., 2005; Mochizuki et al., 2006; Koga et al., 2008). Another attractive characteristic of synovial-resident MSCs is that they have direct access to the synovial fluid, which in turn gives facile migratory access to the superficial layer of cartilage. Early OA-associated damage occurring in the superficial cartilage layers create an anatomic challenge for bone marrow-resident MSCs to reach the site of injury; as it requires their migration through the deeper, undamaged cartilage layers to reach the site in need of repair (McGonagle et al., 2017). Synovial MSCs, on the other hand, would have direct access to the site of injury *via* the articular space in order to initiate repair. This repair pathway was supported by experiments in a canine model showing that synovium-derived MSCs are able to adhere to the areas of cartilage injury (Wood et al., 2012).

Perhaps the most obvious endogenous progenitor cell population for cartilage repair is that which resides within cartilage itself, as recently reviewed by Rikkers et al.( 2022). Termed articular cartilage-derived progenitor cells (ACPCs), these cells most likely reside in the superficial zone in healthy cartilage and will migrate toward the sites of cartilage injury (Grogan et al., 2009; Williams et al., 2010). These cells show similar markers to those of MSCs (CD90, CD105, CD73, and CD166) (Rikkers et al., 2022), possibly distinguished phenotypically by an increased expression of CD44 and enhanced expression of fibronectin and integrin-α5β1 (Dowthwaite et al., 2004; Levato et al., 2017), suggesting a unique progenitor cell population. Correspondingly, OA joint-derived ACPCs were shown to form more colonies *in vitro* compared to those from healthy human cartilage, suggesting greater proliferation capacity with increasing OA severity (Wang et al., 2020; Rikkers et al., 2022). An increase in progenitor markers, such as CD271 (Wang et al., 2020), CD105 (Zhang et al., 2016), and VCAM (Grogan et al., 2009) at sites of trauma or in OA cartilage were also observed. Though these data suggest the involvement of ACPCs in cartilage repair, *in vivo* models outlining corresponding mechanisms are lacking, leaving the role(s) of these progenitor cells in cartilage repair and homeostasis unclear at this time (Rikkers et al., 2022).

**FIGURE 1:**
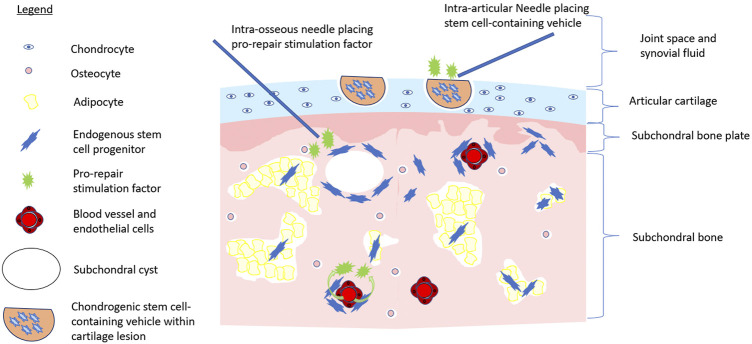
Multistep approach to stem cell-initiated cartilage repair. Image depicts the application of chondrogenic stem cell-containing vehicle (e.g., hyaluronic acid injectate) to a cartilage lesion *via* intra-articular injection while pro-repair stimulation factors are applied to intra-articular space, *via* intra-osseous application, or systemically *via* the bloodstream to activate endogenous repair of the OA joint.

One major hurdle faced by relying on endogenous MSCs to repair cartilage is enhancing their repair capacity. Clearly, the phenotype/reparative potential of these cells in OA patients is inadequate to halt disease progression. Causative factors include irreversible factors such as age. Bone marrow-resident MSCs decline functionally with age (Sethe et al., 2006; Oh et al., 2014), a distinct disadvantage for those with OA, which tends to occur later in life (Felson, 2004). As well, cultured MSCs derived from an inflammatory joint environment have reduced chondrogenic potential *in vitro* (Jones et al., 2010b). *In vivo*, the number of MSCs in magnetic resonance imaging (MRI)-determined bone marrow lesions is five-fold greater than non-bone marrow lesion (Campbell et al., 2016); from a functional perspective; however, these MSCs demonstrated reduced proliferative and osteogenic functional capacity, possibly due to cellular fatigue while residing in a chronically damaged trabecular bone niche (Campbell et al., 2016). Restoring the functional capacity of MSCs or ACPCs at the site of tissue damage could provide a large number of repair cells at the site of injury. With respect to cartilage, regrowth following procedures such as microfracture or bone drilling (albeit often suboptimal fibrocartilage) indicates that a population of bone marrow-resident cells exist within the bone marrow that can repair damaged cartilage (Palmer et al., 2019; Zamborsky and Danisovic, 2020). Enhancing the repair capacity of these cells through the intra-osseous application of growth factors or other molecular interventions may represent a viable treatment opportunity (Delgado et al., 2019) (Figure 1). The application of chemotactic factors to the site of injury to augment the recruitment of endogenous MSCs, as well as other cells involved in the cartilage repair and local immune suppression, could be combined with approaches that enhance the chondrogenic function (Song et al., 2020a). Intra-articular treatment targeting synovium-resident MSCs to enhance their chondrogenic capacity and improve their ability to repair cartilage in an inflammatory microenvironment, or the image-guided placement of intra-defect biomaterials that release these factors to reparative cells upon their migration to the cartilage lesion, may be a less invasive option than the intra-osseous treatment of bone marrow-resident MSCs (Figure 1). Strategies such as arthrocentesis that evacuate the pro-inflammatory synovial fluid of the OA joint, followed by the replacement of the synovial fluid with a pro-chondrogenic cellular and molecular cocktail would be ideal. These treatments, in combination with biomechanical interventions that unload the affected joint compartments such as joint distraction or tibial osteotomy, may further help reduce the hostile microenvironment of the OA joint and prevent further direct mechanical injury (McGonagle et al., 2017; Jansen et al., 2021). Such a combination of interventions could provide a better opportunity for tissue-resident MSCs to repair the cartilage damage, than simply relying on cellular function alone. In summary, our understanding of how endogenous MSCs repair or support the repair of damaged tissue, the effect of the local niche, and potential supporting roles of other cell populations in the OA joint is lacking. As a result, strategies utilizing the recruitment and stimulation of endogenous MSCs to repair OA-associated cartilage injury have yet to emerge. Consequentially, many have turned toward the use of exogenous or transplanted MSCs for cartilage repair.

## Cell-Based Therapies for Osteoarthritis

Although DMOADs for OA are lacking, cell-based therapies have shown promise in reversing the symptoms and structural alterations of OA (Koelling and Miosge, 2009). Given the chondrogenic potential of MSCs, these cells quickly emerged as candidates. Their enhanced ability to differentiate toward chondrogenic lineages under low oxygen tension (Merceron et al., 2010) is also an advantage, due to the avascular and hypoxic nature of cartilage tissue (Goldring and Goldring, 2016). Cell-based therapies currently proposed for OA include autologous cultured chondrocyte transplantation, co-culture and transplantation of MSCs with chondrocytes or hematopoietic-lineage cells, 3D-MSC cultures, or transplantation of MSC-laden scaffolds made of hyaluronic acid, or other synthetic derivatives (Mamidi et al., 2016; Brittberg et al., 1994). In an effort to reduce heterogeneity in therapeutic MSC populations, the International Society for Cell and Gene Therapy (ISCT) developed a set of minimal criteria to define the MSC phenotype, including adherence to plastic, specific antigen expression (e.g., CD73, CD90, and CD105), and multipotent differentiation potential (Dominici et al., 2006). Early phase I–II clinical trials showed improvement in pain and function following the intra-articular application of MSCs into OA-affected knees (Soler et al., 2016; Jo et al., 2014); however, despite these encouraging findings, exogenous MSCs have yet to emerge as a mainstream player for OA treatment. The drawbacks of MSC therapies include the necessity for cell culture, loss of differentiation capacity *ex vivo* or with multiple culture passages, and reduced or halted cellular division after multiple population doublings (Mamidi et al., 2016; Jones and Yang, 2011). Such heterogeneity exists in studies using various animal models of OA in preclinical studies (Cope et al., 2019; Wang et al., 2022), as well as clinical trials. Despite these limitations, several clinical trials have recently reported on the efficacy of exogenous MSCs to regenerate cartilage in OA (Table 1) (Emadedin et al., 2018; Kuah et al., 2018; Freitag et al., 2019; Khalifeh Soltani et al., 2019; Lee et al., 2019; Lu et al., 2019; Matas et al., 2019; Shapiro et al., 2019; Anz et al., 2020; Yang et al., 2022). Across these trials, methodologic heterogeneity has hindered a standardized approach to MSC-based therapies, including the use of host source (allogeneic vs. autologous), tissue source (bone marrow, adipose, umbilical cord, and placenta), injectate (tissue concentrates vs. isolated MSCs), whether they were expanded *in vitro* prior to injection, the dosage used, and the delivery method (e.g., image-guided or not). In addition, not all clinical studies characterized the stem cells being injected, for example, using the ISCT minimal criteria for MSCs (Dominici et al., 2006), making it difficult to ensure what had actually been injected into participants’ joints and impeding the reproducibility of results across studies. Several systematic reviews evaluating the overall efficacy of MSCs for the treatment of OA have been conducted (Kim et al., 2019a; Ha et al., 2019; Song et al., 2020b; Kim et al., 2020) or are underway (Whittle et al., 2019). Although meta-analyses have shown a benefit in pain reduction following MSC intra-articular injection in OA, there is little data supporting their effectiveness for cartilage regeneration. The strength of the evidence supporting their use in clinical practice is, therefore, limited (Kim et al., 2019a; Ha et al., 2019; Song et al., 2020b; Kim et al., 2020). Indeed, many regulatory and scientific bodies, including the United States Food and Drug Administration and Health Canada, have issued position statement warning against the clinical use of these unproven stem cell (SC) therapies for cartilage repair (Marks et al., 2017; CDA, 2019; ISSCR, 2021).

**TABLE 1:** Summary of described clinical trials.

Trial (country)	Sample size	Stem cell source	MSC characterization and laboratory processing	Control	Clinical outcome(s)	Cartilage recovery outcome
Emadedin 2018 (Iran)	47	Autologous BM	ISCT criteria tissue culture expansion	Saline	Pain (VAS) function (WOMAC)	NI
Kuah 2018 (Aus)	21	Allogeneic adipose	No characterization tissue culture expansion	Culture media	Pain (VAS) function (WOMAC)	MRI (MOAKS)
Freitag 2019 (Aus)	30	Autologous adipose	ISCT criteria tissue culture expansion under hypoxic conditions	Usual care	Pain (NRS) function (KOOS)	MRI (MOAKS)
Khalifeh soltani 2019 (Iran)	20	Placenta	No characterization tissue culture expansion	Saline	Pain (VAS) function (KOOS)	MRI (cartilage thickness)
Lee 2019 (Korea)	24	Autologous adipose	Code of federal regulations	Saline	Pain (VAS) function (WOMAC)	MRI (cartilage depth—Noyes grading)
			Characterization tissue culture expansion			
Lu 2019 (China)	53	Autologous adipose	ISCT criteria tissue culture expansion	HA	Pain (VAS) function (WOMAC)	MRI (cartilage volume)
Matas 2019 (Chile)	29	Umbilical cord	ISCT criteria tissue culture expansion	HA	Pain (VAS) function (WOMAC)	MRI (WORMS)
Shapiro 2019 (USA)	25	Autologous BM	ISCT criteria no processing	Saline	Pain (VAS)	MRI (Mean T2 values)
Anz 2020 (USA)	90	Autologous bone marrow	No characterization no processing	PRP	Pain (WOMAC) function (WOMAC)	NI
Yang 2022 (Korea)	176	Umbilical cord	Code of federal regulations	BMAC	Pain (IKDC) function (KOOS)	Arthroscopy (ICRS)

Aus, Australia; BM, bone marrow; BMAC, bone marrow aspirate concentrate; HA, hyaluronic acid; IKDC, International Knee Documentation Committee questionnaire; ICRS, International Cartilage Repair Society score; KOOS, knee injury and osteoarthritis outcome score; MOAKS, MRI osteoarthritis knee score; MRI, magnetic resonance imaging; NI, not included; NRS, numeric rating scale; PRP, platelet-rich plasma; USA, United States of America; VAS, visual analogue scale; WOMAC, Western Ontario and McMaster Universities osteoarthritis index; WORMS, whole-organ magnetic resonance imaging score.

Despite the aforementioned caveats, clinical studies evaluating MSCs for cartilage repair continue (Clinicaltrial.gov, 2022). Factors such as ease of access, low likelihood of side effects, and potential immune-suppressing characteristics are attractive (Whittle et al., 2019). MSCs also present the opportunity to use allogenic sources, suggesting the possibility of an “off-the-shelf” formulation produced through a standardized good manufacturing process (Sanz-Nogués and O'Brien, 2021). With these attractive features in mind, researchers have sought to enhance MSC function *in vivo*. Tissue engineering strategies began in the 1990s using scaffolds and matrices to maintain MSCs at the site of injury and potentiate their repair capacity (Mandrycky et al., 2016; Kim et al., 2019b). Three-dimensional encapsulating matrices could safely deliver MSCs to the site of cartilage injury and provide a biologically optimal milieu for MSCs to repair tissue, protecting them from the hostile inflammatory OA joint environment (Kim et al., 2019b). Growth and differentiation factors could be encapsulated within the matrix, providing stimulus toward chondrogenic differentiation (Kim et al., 2019b). Biomaterials using the proteins fibrin and collagen, or polysaccharides such as hyaluronic acid and agarose have the advantage of being derived from endogenous materials, having good biocompatibility and being biodegradable (Li et al., 2015a). Alternatively, synthetic biomaterials such as polylactic acid, polyglycolide, and polyethylene glycol have the advantage of improved mechanical strength and are easier to mold; for example, to match the shape of a cartilage lesion (Matai et al., 2020). In addition, synthetic biomaterials are immunologically-neutral, are not associated with the risk of transmitting pathogens, and have modifiable chemical and mechanical properties as well as the rate of degradation (Mandrycky et al., 2016; Kim et al., 2019b; Matai et al., 2020). Clinical trials have mainly utilized natural biomaterials such as fibrin, and most studies using synthetic materials were performed in animal models. Like MSCs, the ideal scaffold would be available in a standardized off-the-shelf format.

Culture-expanded MSCs may exert a therapeutic effect through immune modulation and *via* trophic actions on local joint cells through secreted factors without directly participating in new cartilage formation (Mak et al., 2016; Zhang et al., 2022). Consequently, non-cellular biologically-based strategies harnessing the MSC secretome have been explored for cartilage and bone repair (Kalluri and LeBleu, 2020; To et al., 2020; Zhang et al., 2022). MSC-derived extracellular vesicles (MSC-EVs) are MSC-produced nanovesicles ranging from 10 nm to several μm in diameter that contain components such as messenger RNA, microRNA, lipids, and bioactive proteins that produce regenerative paracrine effects within damaged tissue (To et al., 2020). Compared to MSCs, MSC-EVs have the advantage of low toxicity and immunogenicity with repeated transplantation and can be stored for potential off-the-shelf applications (To et al., 2020). A systematic review evaluating the use of MSC-derived extracellular vesicles (MSC-EVs) in preclinical studies for cartilage regeneration showed reduced cartilage loss across a variety of animal models of OA (To et al., 2020). MSC-EVs have been evaluated in preclinical studies for their potential toward bone healing (Kirkham et al., 2021), and a systematic review by Kirkham et al. (2021) reported a promising potential (Kirkham et al., 2021). Though the review focused on fracture healing, pathologic bone changes in the osteochondral unit are well-characterized as a part of OA progression, suggesting that this type of treatment could someday have a role in treating both cartilage and bone-related OA alterations.

In summary, despite important advances in MSC therapy for cartilage regeneration over the last decade, the hunt is still on for the optimal regenerative cell-based approach to repair the cartilage damage associated with OA. In pursuit of the ideal approach, we look to lessons learned from the successes seen in other fields of regenerative medicine, as well as at emerging discoveries of novel chondrogenic stem cell populations, that can be applied to the treatment of OA.

## Hematopoietic Stem Cell Therapy: The Importance of Tissue Microenvironments and Parallels for Cartilage Regeneration

Transplantation of donor-derived hematopoietic stem and progenitor cells to regenerate the hematopoietic system has a long and established history that provided a starting point for future generations of cell therapies (Passweg et al., 2021). Cell product characterization ensured an adequate dose and viability of blood-forming stem cells for the recipient (Gauntner et al., 2021). Extensive regulations and standards protect donors and patients and ensure optimal outcomes following allogeneic transplantation. The processes, standards, and regulations that guide the procurement and transplantation of hematopoietic cell products can be leveraged for other cell-based therapies. The use of autologous cells can also rescue hematopoiesis in recipients following the high-dose chemotherapy with lower risks of transmission of infections, and avoids issues such as graft-versus-host disease. Whether allogeneic or autologous cells are used is an important aspect of cell collection and characterization. The cells from young allogeneic donors can reduce transplant complications and improve survival compared with cells from older allogeneic donors (Shaw et al., 2018). The more robust regenerative capacity of younger cells likely accounts for the improved outcomes. Autologous cells in patients who suffer from disease and/or its treatment may yield cell products with compromised function (Choudhery et al., 2012; Yang et al., 2019) and this should be considered in applications such as cartilage regeneration for OA.

A critical aspect of successful hematopoietic engraftment following transplantation relates to the function and status of the bone marrow microenvironment (Morrison and Scadden, 2014). Recipient age, prior therapy, and disease-induced changes in the marrow microenvironment can impair hematopoiesis. MSCs, a chief component of the marrow microenvironment, that are derived from patients with acute myeloid leukemia are abnormal with skewed differentiation potential and reduced ability to support normal hematopoiesis (Chandran et al., 2015; Le et al., 2016). Understanding the tissue microenvironment will be paramount in cell therapies for cartilage regeneration. Strategies restoring the health of the tissue microenvironment may augment the success of cell-based therapies. Exercise, for instance, was shown in a mouse model to accelerate hematopoietic engraftment following transplantation (De Lisio et al., 2013) and nutritional status including the essential amino acids is crucial for robust hematopoiesis (Wilkinson et al., 2018).

A global network of registries of healthy volunteer donors exists to facilitate the collection of blood stem cells to support unrelated allogeneic hematopoietic transplantation. The process of identifying HLA-compatible donors who are healthy and free of transmissible disease is well-established through the World Marrow Donor Association and its connected network of international registries and collection centers (Bochtler et al., 2011). Whether these donors could provide cells and tissues to support other forms of cellular therapy is intriguing. In a recent survey of registrants on the Stem Cell Registry at Canadian Blood Services, many registrants were willing to donate cells for uses other than blood cell transplants (Liao et al., 2020).

Leveraging the clinical experience from hematopoietic transplantation in the areas of product characterization and donor cell procurement may accelerate the translation of stem cell therapy to cartilage degeneration in OA.

## Muscle Stem Cell Therapy

The development of cell-replacement therapies for the treatment of muscle wasting diseases has received much attention (Dao et al., 2020). Most efforts focused on the muscle-resident stem cell, termed satellite cells, juxtaposed between the basal membrane, and muscle fiber (Aziz et al., 2012). Since their discovery in the early 1960s (Katz, 1961; Mauro, 1961), considerable knowledge was gained on the role of satellite cells in muscle repair, the influence of the niche in which they reside, as well as the role of other niche-supporting cells (Sousa-Victor et al., 2021). These critical aspects of the repair process are less-well described for cartilage repair (McGonagle et al., 2017). For example, in normal resting muscle, the majority of satellite cells are maintained in a long-lived Pax7-expressing quiescent (G0 reversible arrest) state (Aziz et al., 2012; García-Prat et al., 2016). To ensure tissue homeostasis, satellite cells will repair small myofiber defects by re-entering the cell cycle, undergoing a single asymmetric cell division that generates one differentiating daughter cell that will contribute to the myofiber and one daughter stem cell to replenish the stem cell pool (Aziz et al., 2012; Sousa-Victor et al., 2021). Only after enough progenitor cells have been made to repair the myofiber will a small number of progenitors return to the quiescent state to repopulate the stem cell niche (Cutler et al., 2021; Robinson et al., 2021).

Myogenic regulatory factors (MRFs) contribute to establishing the myogenic cell identity, to the subsequent differentiation and formation of muscle fibers during muscle regeneration in postnatal life (Sousa-Victor et al., 2021). Molecular mechanisms governing the transition between quiescence and activation have been studied and include transcriptional and post-translational, epigenetic (Sousa-Victor et al., 2021), as well as metabolic and proteostatic regulation (Sousa-Victor et al., 2021). In addition, several extrinsic factors, including epidermal growth factor (Roe et al., 1989), hepatocyte growth factor (Miller et al., 2000), angiopoietin 1 (Abou-Khalil et al., 2009), nitric oxide (Wehling et al., 2001), fibroblast growth factor (Bischoff, 1986), and insulin-like growth factor (Allen and Boxhorn, 1989), are known to modulate satellite cell quiescence, activation, expansion, self-renewal, and differentiation (Kuang et al., 2008; Aziz et al., 2012). Further modulation of satellite cells occurs through other cells within the satellite cell niche, including macrophages, neutrophils and other white blood cells, fibroadipogenic progenitors (FAPs), and endothelial cells (Sousa-Victor et al., 2021). The satellite cell crosstalks with other constituents of the niche through signaling pathways such as TGFβ, Notch, and Wnt further refines the satellite cell function and fate (Sousa-Victor et al., 2021).

This breadth of data regarding the phenotype, function, and niche of satellite cells has provided a foundation of knowledge toward stem cell therapy for muscle-related conditions, including age-related sarcopenia (García-Prat et al., 2016; Lukjanenko et al., 2019) and muscular dystrophies (Périé et al., 2014; Campbell and Puymirat, 2021). Like those faced by MSC therapies, ongoing challenges with the use of satellite cell-derived myoblasts include cell culture-passaging limitations that restrict their expansion potential *in vitro*, and the development of specific cell differentiation protocols (Chal and Pourquié, 2017; Magli et al., 2017; Xi et al., 2017). The continuously evolving knowledge of satellite cell repair mechanisms, the role of their niche, and niche-supporting cells provides great optimism for their therapeutic use for muscle disease in the future. Such a foundation of knowledge may contribute to the stem cell regenerative approach to cartilage repair in OA.

## Future Avenues for Stem Cell Therapy for Cartilage Regeneration

Stimulating native articular stem cells to enhance their ability to repair cartilage *in vivo* constitutes a viable avenue for future research. Among MSCs, a significant amount of heterogeneity exists (Jones and Schafer, 2015). Factors such as culture age, donor sex, and health status play a role in the MSC function and surface receptor expression (Murphy et al., 2002; Baxter et al., 2004; Astudillo et al., 2008; Zhen et al., 2013; Jones and Schafer, 2015). Of these factors, the niche from which MSCs are isolated is believed to have a decisive influence on their function and differentiation capacity (Risbud et al., 2006; Jones and McGonagle, 2011; de Sousa et al., 2014; Jones and Schafer, 2015). Bone marrow-derived MSCs are believed to have a more optimal capacity for chondrogenic differentiation than those derived from adipose tissue, while adipose-derived MSCs have a higher proliferative capacity (Li et al., 2015b). Even among bone-derived MSCs, the particular bone and the topographic region, therein, can influence differentiation capacity and surface marker expression (Risbud et al., 2006; Ackema and Charite, 2008; Tormin et al., 2011; Jones and Schafer, 2015). Therefore, selecting an appropriate endogenous stem cell target and niche to assist in OA joint repair will be a crucial factor toward positive outcomes. Similarly, the tissue of origin of exogenous MSCs transplanted into the site of cartilage injury will also be a primary factor toward successful cartilage regeneration. Interestingly, while studies have compared stem cell chondrogenic capacity from different niches *in vitro* (Mochizuki et al., 2006; Koga et al., 2008), and studies have compared stem cells to implanted chondrocytes (Nejadnik et al., 2010) and bone marrow concentrate (Yang et al., 2022) *in vivo*, we are not aware of any *in vivo* studies directly comparing chondrogenic repair capacities of different stem cell populations derived from different tissue sources. To date, clinical trials evaluating MSCs have shown some evidence of reducing OA-associated pain and can produce cartilage *in vitro*, but there is little evidence that current MSC treatment regenerates damaged cartilage *in vivo*. In sum, the hunt is still on for the most potent stem cell population capable of mediating cartilage repair *in vivo*. This begs the question: where would such a population exist?

Beyond MSCs derived from adipose sources and articular tissues, other potential candidates for cartilage repair are also being pursued. Dental-derived MSCs, such as dental pulp stem cells (DPSCs) originating from neural crest mesenchyme in the dental pulp, can be extracted with minimal donor site morbidity (Ibarretxe et al., 2012; Lo Monaco et al., 2020; Li et al., 2021). The dental pulp provides a protective environment for DPSCs during a person’s lifetime, preserving their stem cell capacity (Fernandes et al., 2018). *In vitro*, DPSCs can be differentiated into cartilage-producing cells and can secrete several chondrogenic growth factors (Bronckaers et al., 2013; Ahmed Nel et al., 2016). Like bone-derived MSCs, they also have immunomodulatory capabilities that may be beneficial in the inflamed OA joint (Li et al., 2014; Lo Monaco et al., 2020). Preclinical models evaluated DPSC chondrogenic capacity. Lei et al. (2014) transplanted human DPSC cell pellets into the dorsal surface of immunodeficient mice where they maintained their chondrogenic capacity (Lei et al., 2014). Lo Monaco et al. (2020) showed an improved *in vitro* pro-survival effect when immature murine chondrocytes were cultured with DPSC-conditioned media (Lo Monaco et al., 2020). Mata et al. (2017) evaluated the ability of DPSCs to repair osteochondral defects in rabbits using alginate matrix-embedded DPSCs and alginate-embedded rabbit chondrocytes. Compared to controls, both rabbit chondrocytes and human DPSCs showed an improved quantity of *in vivo* cartilage regeneration and collagen fiber alignment (Mata et al., 2017). Fernandes et al. (2018) demonstrated that the DPSCs in a biomaterial scaffold showed a thicker deep layer of cartilage with less fibroblastic tissue, as compared to scaffold-alone (Fernandes et al., 2018). Overall, though DPSCs remain in the preclinical stage of experimentation for cartilage regeneration, they have shown potential to repair cartilage lesions *in vivo* and to promote native chondrocyte survival. These encouraging data may eventually benefit patients with OA.

The growth plate is the site of long bone growth in youth, through the process of endochondral bone growth (Kronenberg, 2003; Berendsen and Olsen, 2015). This process involves the longitudinal growth and ossification of a cartilage matrix, which is initiated by the action of chondrocytes in the *proliferative zone* of the growth plate (Koelling et al., 2009). These, in turn, are derived from small and relatively inactive stem cells located in the *reserve zone* close to the secondary ossification center (Koelling et al., 2009). Endochondral ossification and bone growth, therefore, rely on a yet-uncharacterized population of undifferentiated skeletal stem cells (SSCs) with highly robust chondrogenic functional capacity that resides in a protected niche within the growth plate (Chan et al., 2015). In 2015, Chan isolated SSCs from the femoral head growth plate of mice (Yin et al., 2013). The characterization of these skeletal stem cells showed a robust ability to differentiate *in vitro* to chondrocytes, osteoblasts, or bone marrow stromal cells. Although the chondrogenic potential of these murine SSCs was not directly compared to that of MSCs, studies from muscle (and other tissues) suggest that tissue-embedded stem cell populations are more efficient at repairing damaged tissue compared to MSCs (i.e., MSCs sacrifice efficiency for versatility while tissue-specific stem cells are more efficient at repairing, but show limited versatility) (Jankowski et al., 2002; Chan et al., 2018). In 2018, a human SSC population demonstrating self-renewal and multilineage differentiation to the bone, cartilage, and stroma was isolated from the growth plate of human embryonic femoral bones (Murphy et al., 2020). Within this growth plate population, several subpopulations were described that were able to produce cartilage *in vitro*, identified by surface marker expression PDPN^+^CD146^−^. The cells that were CD146^+^, a marker commonly associated with MSCs, displayed reduced colony size and frequency compared to PDPN^+^CD146^−^ SSCs, as observed by light microscopy and flow cytometry (Murphy et al., 2020). As well, two of the three chondrogenic SSC subpopulations did not express CD73, a marker included in the ISCT MSC definition, further suggesting these cells are not MSCs and are indeed a distinct and functionally unique stem cell population. The SSC with greatest functional diversity, including high chondrogenic potential, was identified as PDPN^+^CD146^−^CD73^+^CD164^+^ with the highest expression of these marker transcripts isolated from the proliferative and pre-hypertrophic zone of fetal bones (Murphy et al., 2020). The authors developed a monocyte-derived induced pluripotent stem cell (iPSC) line that also expressed PDPN^+^CD146^−^CD73^+^CD164^+^ and produced cartilage *in vivo,* an important finding that would allow laboratory-based production of a cartilage-generating cell line not requiring access to fetal tissue (Murphy et al., 2020). We are unaware of clinical trials evaluating SSCs applied exogenously for cartilage repair.

In line with enhancing the endogenous stem cell population toward repairing cartilage defects *in vivo*, Murphy et al. evaluated the response of SSCs to microfracture in both the distal femur of mice that had undergone the destabilization of the medial meniscus (a well-described model of OA), as well as human fetal phalangeal bone (Csobonyeiova et al., 2021). Following the articular surface microfracture in mice, the authors identified a proliferation of SSCs; however, the resulting regenerative tissue appeared to be morphologically heterogeneous, containing both fibrotic and chondrogenic tissue (Csobonyeiova et al., 2021). The addition of a hydrogel containing BMP2 and a VEGF antagonist enhanced cartilage tissue formation that approximated the structural properties of native cartilage. Similar results were achieved following the microfracture of the human phalangeal articular surface with BMP2 and anti-VEGF treatment, suggesting that endogenous SSCs could be stimulated toward cartilage regeneration following microfracture intervention. Whether such potent cartilage regeneration can be achieved in adult OA-affected articular remains to be determined.

Recently, research has emerged evaluating the differentiation of iPSCs toward chondrocytes for the purpose of treating OA (Takahashi et al., 2007). iPSCs were initially generated from somatic cells by the viral transfection of key reprogramming factors (Oct3/4, Sox2, c-myc, and Klf4) into the donor cells (Koyama et al., 2013). Like embryonic stem cells, iPSCs are pluripotent and have similar cell morphology, gene expression, and proliferation capability; however, iPSCs are derived from somatic cells, avoiding the ethical issues related to collecting cells from embryos (Takahashi et al., 2007). iPSCs may be differentiated toward the chondrocyte lineage using media supplemented with growth factors such as TGF-β, BMP, WNT3A, and FGF-2 (Takahashi et al., 2007). The four main chondrogenic differentiation approaches used to date include 1) the generation of MSC-like iPSCs with further differentiation into chondrocytes (Takahashi et al., 2007; Qu et al., 2013; Nejadnik et al., 2015); 2) co-culture of iPSC-derived MSCs with primary chondrocytes (Takahashi et al., 2007; Rim et al., 2018); 3) through the formation of three-dimensional cellular aggregates (Takahashi et al., 2007; Yamashita et al., 2015); and 4) culturing of iPSCs in a series of media which mimics physiological developmental pathways (Takahashi et al., 2007; Murphy et al., 2018). Using the appropriate cell culture conditions, iPSCs can also be differentiated towards potent chondrogenic progenitors, such as SSCs (Murphy et al., 2020), suggesting that they could generate a pool of precursors mimicking the functionality of a chondrogenic stem cell population. The drawbacks of iPSCs, however, includes genomic instability, difficulties in obtaining uniform mature cell populations, and tumor formation, owing to the risks of insertional mutagenesis and reactivation of transgenes caused by the integration of the viral genome used to create these cells (Takahashi et al., 2007; Martel-Pelletier et al., 2016). Strategies for overcoming these issues, as well as improving differentiation protocol efficiency of the phenotype of the ideal stem cell population for cartilage regeneration is an ongoing and exciting area of research (Takahashi et al., 2007; Martel-Pelletier et al., 2016).

## Conclusion

**FIGURE 2:**
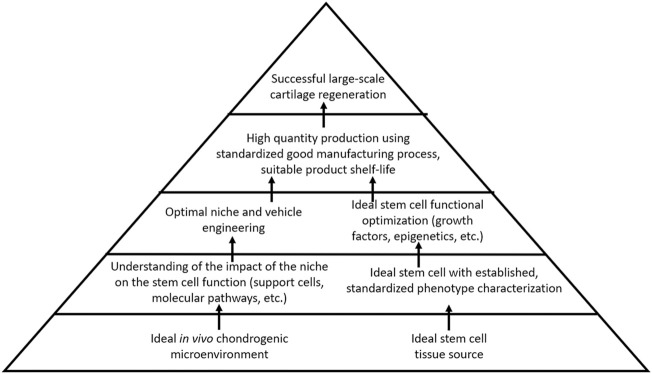
Suggested directions for cell-based therapy research for cartilage regeneration.

Current strategies to regenerate cartilage in the OA joint are limited to surgical interventions that may fail or yield fibrocartilaginous tissue with insufficient biomechanical properties for joint load distribution. Furthermore, such surgical treatments are less ideal for larger, non-focal areas of cartilage loss, as seen in the later stages of OA. Regenerative medicine thus remains an attractive option for OA treatment. Although stem cell therapy holds promise for cartilage regeneration in OA, safe and reliable strategies to either optimize endogenous stem cells toward cartilage repair, to apply exogenous stem cells into damaged joints, or both simultaneously, have yet to emerge. The research study has emphasized the importance of the stem cell source niche, which proffers the characteristics that stem cells bring to the table with respect to their regenerative capacity. Despite this, however, there remains a lack of standardization across clinical studies regarding stem cell tissue source and phenotypic characterization. A well-characterized cell product that can be reproducibly isolated or manufactured, and that can reliably produce healthy cartilage in the avascular cartilage microenvironment—that is, standardization of stem cells used for treatment and evidence-based outcome measures—will be an essential factor for moving the field of cartilage regeneration forward. The effective means of contending with the catabolic microenvironment of the OA joint and maintaining cellular chondrogenic functional capacity in a region primed toward cartilage destruction will also be an important challenge to overcome. The lessons from other areas of regenerative medicine can be applied to the field of cartilage regeneration. Hematopoietic therapies benefit from a deep understanding of the impact of the microenvironment on stem cell activity, not only the stem cells, but also the intricate role played by other support cells within the hematopoietic niche. A well-established international infrastructure responsible for thoroughly characterizing, standardizing, and distributing hematopoietic stem cells represents a major advantage. The field of muscle regeneration also takes the advantage of decades of data describing the mechanism of regeneration as well as the impact on the microenvironment on the satellite cells, additionally benefiting from a strong understanding of molecular pathways by which satellite cells develop, replicate, and respond to external function-altering stimuli. As compared to these other areas of regenerative medicine, our current understanding of the optimal cartilage-producing stem cell, its niche, the identity and role of niche-supporting cells, and the molecular mechanisms governing functional potential remains limited. Further research is needed to address these fundamental factors (Figure 2).

**FIGURE 3:**
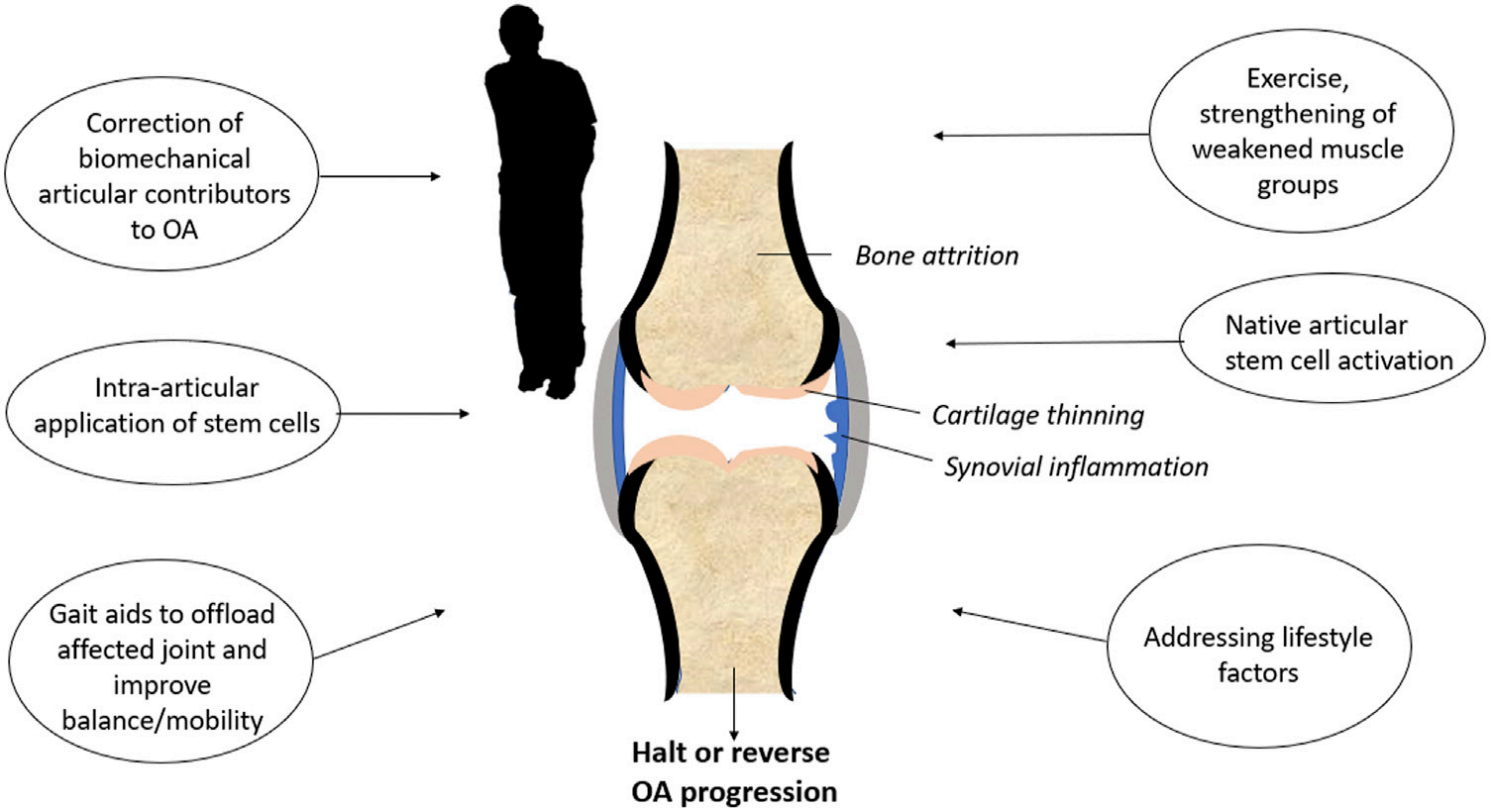
Multimodal treatment of the osteoarthritis-affected knee.

Even though stem cell therapy will likely 1 day become a valuable tool for cartilage regeneration, OA remains a whole-joint disease (Martel-Pelletier et al., 2016). Articular tissues including bone, synovium, and the joint capsule are all affected by OA, as well as peri-articular tissues such as muscle (Goldring and Goldring, 2016; Martel-Pelletier et al., 2016). Protocols, such as stem cell injections used in isolation, are unlikely to halt or reverse OA if the numerous factors contributing to joint degeneration are not addressed. Multimodal approaches (Figure 3), such as compartmental or total joint unloading to remove excess biomechanical stress, physiotherapy for muscle reconditioning, and lifestyle changes such as weight control and regular exercise will give the ideal stem cell population a fighting chance to regenerate the cartilage in people suffering from OA.
